# Emotional ecosystems: Understanding the relationship between family interactions and anxiety among cancer caregivers

**DOI:** 10.1017/S147895152400213X

**Published:** 2025-01-30

**Authors:** Keisha White Makinde, Jacquelyn J. Benson, Kyle A. Pitzer, Maysara Mitchell, Debra Parker Oliver, George Demiris, Karla T. Washington

**Affiliations:** 1 Washington University in St. Louis School of Medicine, Saint Louis, MO, USA; 2 Goldfarb School of Nursing at Barnes-Jewish College, Saint Louis, MO, USA; 3 University of Pennsylvania School of Nursing, Philadelphia, PA, USA; 4 University of Pennsylvania Perelman School of Medicine, Philadelphia, PA, USA

**Keywords:** Caregivers, palliative care, mental health, family support, neoplasms

## Abstract

**Objectives:**

Recent studies have challenged the assumption that families are invariable sources of support for cancer caregivers, noting that relationships with family members can have both positive and negative effects on caregiver well-being. This study expands upon prior literature to examine the relationship between cancer caregivers’ perceptions of the quality of their family interactions and their symptoms of anxiety.

**Methods:**

We employed secondary analysis of baseline data from a multisite randomized clinical trial of an intervention for cancer caregivers conducted at 3 large academic palliative care clinics. We performed linear regression analyses to analyze the relationship between caregivers’ perceptions of the quality of their family interactions and their symptoms of anxiety; additional models were estimated to further characterize this relationship with the addition of relevant covariates: race, ethnicity, sex, marital/relationship status, relationship to patient, employment status, household income, and perceived social support received from friends and significant others. We also conducted a sub-analysis of data provided by caregivers who were married or partnered to examine the relationship between their perceptions of the quality of their family interactions and their symptoms of anxiety with relationship satisfaction as a covariate.

**Results:**

Among our analytic sample (*n* = 244), we identified a significant negative relationship between cancer caregivers’ perceptions of the quality of their family interactions and their symptoms of anxiety; this relationship remained statistically significant with the addition of covariates. Relationship satisfaction was not found to be a statistically significant covariate in our sub-analysis of married or partnered caregivers.

**Significance of results:**

Study results provide strong support for the development, testing, and implementation of interventions to improve family interactions as a strategy to reduce caregiver anxiety.

## Introduction

Although rates of cancer diagnosis have remained fairly steady over the past several decades, cancer survivorship is increasing (National Cancer Institute, NIH, DHHS 2023) due to improvements in medical technology and cancer therapies. Alongside many cancer patients are dedicated family caregivers supporting those living with cancer (Family Caregiver Alliance 2016). Family caregivers provide a crucial level of emotional, financial, and instrumental support that the U.S. medical system is not designed to provide. For instance, many caregivers ensure the patient’s prescriptions are available and administered at appropriate times, help with bathing and maintaining hygiene, assist with cooking meals and other household tasks, and communicate with the healthcare team (National Alliance for Caregiving and AARP 2020). Although many caregivers take pride in and receive great personal benefit from caring for a family member (Dsf et al. 2018), caregivers also face significant challenges due to the stress of their role (Janson et al. 2022). Cancer caregivers provide care for an average of 3.9 years, with 39% reporting a high-intensity level of care (National Alliance for Caregiving and AARP 2020). Furthermore, 36% of cancer caregivers report high levels of emotional stress (National Alliance for Caregiving and AARP 2020). Although there is significant heterogeneity in services available to support family caregivers of cancer patients, many are designed to reduce caregivers’ psychological distress, including symptoms of anxiety (Oliveira et al. 2022).

Anxiety – an emotion distinguished by “apprehension and somatic symptoms of tension in which an individual anticipates impending danger, catastrophe, or misfortune” (American Psychological Association 2023; Janson et al. 2022) – has been highlighted as a prevalent experience among family caregivers (Ahmad Zubaidi et al. 2020). When care recipients’ health and functional status are worse, caregivers typically experience more anxiety (De Laurentis et al. 2019). Given the unique stressors of caregiving, it is crucial that caregivers receive adequate supports for their mental health, as untreated anxiety symptoms can lead to a disabling anxiety disorder (American Psychiatric Association 2013). Thus, there is significant interest among cancer researchers and clinicians in identifying appropriate targets for interventions aimed at anxiety reduction.

### The stress buffering hypothesis

First described by Cohen and Wills (1985), the stress buffering hypothesis proposes that social support can protect against the toxic effects of stress. Social support refers to a network of individuals (friends, family, community members, religious leaders, etc) who are available to provide emotional, tangible, informational, or esteem support during times of need or stress. Within the stress buffering hypothesis, social support may prevent one’s appraisal of a potentially stressful experience as a stressor (Cohen and Wills 1985); for instance, for cancer caregivers, this may mean that a supportive family member may help the caregiver reframe their caregiving experience in a positive light, thus preventing appraisal of the situation as stressful and reducing the likelihood of worsened health. Cohen and Wills also propose that social support can be helpful even when an experience is appraised as stressful by helping to avoid maladaptive responses to stress and/or promote one’s use of adaptive coping skills, both of which buffer the stress experience and defend against deteriorated health. In their systematic review of a decade of cancer caregiving studies, Ochoa et al. (2020) found that social support was a frequently investigated construct, with research consistently finding it to be associated with caregiver wellbeing; however, they noted that studies focusing more specifically on caregivers’ perceptions of the quality of their relationships with individuals in their social network were uncommon, highlighting a significant gap in the literature.

### Cancer caregivers’ family interactions

Family interactions are a significant component of family quality of life (Alnahdi et al. 2022; Rettig and Leichtentritt 1999) and can impact caregiver wellbeing and mental health (Rurka et al. 2021). In essence, family interactions refer to the quality of day-to-day interactions one has with their family members. While family interactions can be an important source of support for caregivers (Oliver et al. 2017), they can also introduce additional stress (Benson et al. 2023; Hastert et al. 2020). Cancer caregivers, in particular, report strained family relationships and psychosocial dysfunction (Nissen et al. 2016), challenging romanticized notions of family as an invariable source of social support. For spousal caregivers, there is interrelatedness between caregiver and care recipient needs and distress (Litzelman and Al Nassar 2022), and relationship satisfaction (or dissatisfaction) plays a significant role in quality of life for cancer patients and caregivers (Galbraith 2005). Furthermore, caregivers from marginalized identities including racial minorities and low socioeconomic status may face unique challenges that may be additive stressors, such as experiences of racism and discrimination within the caregiving context and conflicts between upholding African American cultural norms and the norms of a westernized healthcare system (Cothran et al. 2022; Starr et al. 2022).

### Study purpose and hypothesis

Existing evidence highlights the importance of family interactions in caregivers’ lives, yet has failed to adequately examine if and how the quality of those interactions affects caregiver wellbeing. This study sought to address this gap in the knowledge base by investigating the association between perceived quality of family interactions and symptoms of anxiety among cancer family caregivers. We hypothesized that there would be a negative relationship between family interaction quality and anxiety, whereby caregivers who perceived their family interactions as more positive would endorse fewer anxiety symptoms.

## Methods

This study employed a secondary analysis of baseline data available from the ongoing National Cancer Institute-funded multisite randomized clinical trial “Problem-Solving Therapy for Cancer Caregivers” (R01CA258311). Participants were recruited from 3 academic palliative care clinics in the U.S. Midwest (2) and East (1). Eligible participants were adult family caregivers (defined as anyone substantially involved in a patient’s care on an unpaid basis) to adult patients diagnosed with cancer and receiving outpatient palliative care. We excluded from our initial, bivariate analysis participants who did not provide data on perceived quality of family interactions or anxiety symptoms, resulting in a starting analytic sample of 244 caregivers. Additionally, we estimated a model with a subset of married or partnered participants to examine relationship satisfaction as a potential covariate; after removing cases with missing data on perceived quality of family interactions, anxiety symptoms, or any covariate, the sample for this sub-analysis included 173 caregivers. The study protocol was reviewed and approved by the Washington University in St. Louis Institutional Review Board (IRB ID #202104120).

### Measures

#### Explanatory and outcome variables

##### Family Quality of Life in Dementia-Family Interactions subscale

The primary explanatory measure of interest in our models was the Family Interactions subscale of the Family Quality of Life in Dementia scale (FQOL-FI). Development and validation of the full scale are described in detail by Rose et al. (2021). Essentially, the Family Interactions subscale measures the quality of one’s interaction with family members. This measure’s score is the sum of 14 items using a 1–5 Likert-type scale with agreement anchors. These items include statements regarding family interactions, such as: “My family members have the ability to talk openly with each other and discuss difficult issues,” and “My family members show that they love and care for each other.” The Family Interactions subscale does not include items or prompts related to dementia; statements pertain to various aspects of family interactions, such as open communication, addressing challenging topics, and displaying love and care among family members. Scores range from 14 to 70, with higher scores reflecting better perceived quality of family interactions (Rose et al. 2021). The internal consistency reliability for this measure in our sample was very high (Cronbach’s α = .94)

##### Patient-Reported Outcomes Measurement Information System (PROMIS) Short Form v1.0 – Anxiety 8a

The primary outcome in our models was anxiety, measured by the PROMIS Short Form v1.0 – Anxiety 8a (PROMIS, an initiative of the National Institutes of Health that aims to produce rigorously tested patient-reported outcome measures, is described elsewhere (Cheng et al. 2023; Pilkonis et al. 2011). The measure has been validated in numerous populations (de Castro et al. 2020; Purvis et al. 2019), including validation for cancer patients (Cai et al. 2021; Clover et al. 2022; Victorson et al. 2019). Its raw score is the sum of 8 items using a 1–5 Likert-type scale with frequency anchors measuring symptoms of anxiety. Raw scores ranging from 8 to 40 are converted into standardized T-scores, which have a mean of 50 and a standard deviation of 10 in the general U.S. population (Pilkonis et al. 2011). Higher scores indicate more anxiety. The internal consistency reliability within our sample for the raw scores was also high (Cronbach’s α = .92).

#### Covariates

##### Demographics

Participants self-reported the following demographic variables during baseline assessments: age, race, ethnicity, sex, marital/relationship status, relationship to patient, employment status, and household income.

##### Multidimensional Scale of Perceived Social Support

We adapted the Multidimensional Scale of Perceived Social Support (MSPSS) for use in this study. The original scale includes 12 items that assess perceptions of support from family, friends, and significant others (Zimet et al. 1990). For this study, we excluded family items due to overlap with questions in the FQOL-FI. We performed a Kendall’s tau correlation test to assess discriminant validity between the FQOL-FI and adapted MSPSS and found a weak association (τ = .27, *p* < .001), which provides additional evidence that the 2 scales measure distinct constructs. The MSPSS subscales used here focus on perceived support from friends and significant others. By incorporating these into our analysis, we were attempting to delineate the effect of these types of social support from social support received from family members, which was the primary focus of our study. The adapted measure is the sum of 8 items using 1–7 Likert-type scales with agreement anchors. Scores ranges from 8–56. Scale development and psychometric properties are described in detail elsewhere (Dambi et al. 2018; Zimet et al. 1990). The MSPSS indicated very high internal consistency reliability within our sample (Cronbach’s α = .95).

##### Relationship satisfaction

Respondents were asked the following researcher-generated question to ascertain relationship satisfaction: “In general, how satisfied are you in your marriage/current partnership?” Responses were selected from a 5-point Likert-type scale with higher scores corresponding to increased satisfaction.

### Statistical analysis

All statistical analyses were completed using R Statistical Software version 4.2.1 (R Core Team 2021).Prior to testing our hypothesis regarding the relationship between perceived quality of family interactions and anxiety, we examined descriptive statistics for all model variables. We examined mean, standard deviation, median, and range for continuous variables and frequencies for categorical variables. We utilized a block-wise approach to estimate the linear models. The first model in the procedure included only the outcome variable (anxiety symptoms) and perceived quality of family interactions; the second model added demographic and contextual covariates. We also estimated a 3rd model that examined a subsample of married or partnered caregivers with the addition of relationship satisfaction as a covariate (this model also included the covariates included in our second model). For model results, we report the estimates and significance for individual variables and the overall fit of the model to the data. We considered *p* values < .05 as statistical evidence of a relationship between our explanatory variable (perceived quality of family interactions) and covariates and our outcome variable (anxiety symptoms). To determine the results of our hypothesis testing, we used these estimates and their associated *p* values. We also examined model assumptions and diagnostics using a scatterplot for residuals and fitted values, a normal q–q plot, and a scale-location plot. Missing data were addressed via list-wise deletion.

## Results

The sample (*N* = 244) is described in Table 1. Most of the sample identified as White (82%), not Hispanic or Latina/o (96%), and female (65%), with an average age of 55. The largest proportion were married (68%), the spouse of the patient (49%), and employed full-time (43%), with a household income of greater than $70,000/year (53%). The average perceived social support from friends and significant others was high, with a mean of 44.6 (standard deviation [SD] = 10.0) out of a possible 56. The average anxiety score was 56.8 (SD = 9.54), slightly higher than that of the general U.S. population. The average perceived quality of family interactions score was 57.5 (SD = 11.7). The married or partnered subsample included 173 participants; nearly 79% of the married or partnered subsample were very satisfied with their relationship (additional descriptives for the subsample are presented in Table 2). Note this subsample only includes cases with valid data for all subsample variables.

**Table 1.** Sample characteristics

	Overall (*N* = 244)
**Age**	
Mean (SD)	55.0 (15.6)
Median (min, max)	58.0 (18.0, 95.0)
**Race**	
White	200 (82.0%)
Black/African American	22 (9.0%)
Other	17 (7.0%)
Missing	5 (2.0%)
**Ethnicity**	
Not Hispanic or Latina/o	233 (95.5%)
Hispanic or Latina/o	9 (3.7%)
Missing	2 (0.8%)
**Sex**	
Male	82 (33.6%)
Female	159 (65.2%)
Other	1 (0.4%)
Missing	2 (0.8%)
**Marital/relationship status**	
Single, never married	23 (9.4%)
Married	165 (67.6%)
In a committed partnership	32 (13.1%)
Divorced or separated	16 (6.6%)
Widowed	6 (2.5%)
Other	2 (0.8%)
**Caregiver relationship to patient**	
Adult child	60 (24.6%)
Spouse	120 (49.2%)
Ex-spouse	4 (1.6%)
Unmarried partner	18 (7.4%)
Parent	18 (7.4%)
Sibling	12 (4.9%)
In-law	4 (1.6%)
Other family member	8 (3.3%)
**Employment status**	
Unemployed	22 (9.0%)
Employed part-time	25 (10.2%)
Employed full-time	106 (43.4%)
Retired	71 (29.1%)
Other	19 (7.8%)
Missing	1 (0.4%)
**Household income**	
Less than $20,000/year	21 (8.6%)
$20,000–$40,000/year	29 (11.9%)
$40,001–$70,000/year	46 (18.9%)
More than $70,000/year	130 (53.3%)
Missing	18 (7.4%)
**Social support from friends and significant others**	
Mean (SD)	44.6 (10.0)
Median (min, max)	47.0 (8.00, 56.0)
Missing	5 (2.0%)
**Anxiety symptoms**	
Mean (SD)	56.8 (9.54)
Median (min, max)	57.9 (37.1, 83.1)
**Perceived quality of family interactions**	
Mean (SD)	57.5 (11.7)
Median (min, max)	61.0 (14.0, 70.0)

**Note**: SD = standard deviation; min = minimum; max = maximum.

Percentages may not total 100 due to rounding.

**Table 2.** Subsample characteristics (married or partnered caregivers)

	Overall (*N* = 173)
**Age**	
Mean (SD)	55.4 (14.4)
Median (min, max)	58.0 (19.0, 84.0)
**Race**	
White	151 (87.3%)
Black/African American	11 (6.4%)
Other	11 (6.4%)
**Ethnicity**	
Not Hispanic or Latina/o	166 (96.0%)
Hispanic or Latina/o	7 (4.0%)
**Sex**	
Male	64 (37.0%)
Female	109 (63.0%)
**Caregiver relationship to patient**	
Adult child	35 (20.2%)
Spouse	101 (58.4%)
Ex-spouse	1 (0.6%)
Unmarried partner	14 (8.1%)
Parent	10 (5.8%)
Sibling	6 (3.5%)
In-law	3 (1.7%)
Other family member	3 (1.7%)
**Employment status**	
Unemployed	11 (6.4%)
Employed part-time	19 (11.0%)
Employed full-time	83 (48.0%)
Retired	47 (27.2%)
Other	13 (7.5%)
**Household income**	
Less than $20,000/year	11 (6.4%)
$20,000–$40,000/year	18 (10.4%)
$40,001–$70,000/year	33 (19.1%)
More than $70,000/year	111 (64.2%)
**Relationship satisfaction**	
Very dissatisfied	3 (1.7%)
Somewhat dissatisfied	8 (4.6%)
Neither dissatisfied nor satisfied	6 (3.5%)
Somewhat satisfied	20 (11.6%)
Very satisfied	136 (78.6%)
**Social support from friends and significant others**	
Mean (SD)	45.3 (9.00)
Median (min, max)	47.0 (12.0, 56.0)
**Anxiety symptoms**	
Mean (SD)	57.2 (9.24)
Median (min, max)	57.4 (37.1, 83.1)
**Perceived quality of family interactions**	
Mean (SD)	58.1 (11.4)
Median (min, max)	61.0 (18.0, 70.0)

*Note.* Sample includes only cases with valid data on all model variables.

SD = standard deviation; min = minimum; max = maximum.

Percentages may not total 100 due to rounding.

**Figure 1.**
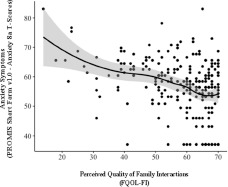
Bivariate relationship between perceived quality of family interactions and anxiety symptoms.

Model results for anxiety symptoms including covariates indicated that perceived quality of family interactions was significantly associated with symptoms of anxiety (*b* = −0.212, standard error [SE] = .059; Table 3). The bivariate relationship is shown in Fig. 1. Estimates suggest that on average, for every 10-point increase in perceived quality of family interactions, anxiety decreased by 2.1 points. This finding was consistent across both models, although the effect slightly diminished when covariates were added. Findings also indicated that age, sex, and marital/relationship status. More specifically, on average, a higher anxiety score was associated with being female and with being married or in a committed partnership compared to being single. Conversely, a lower anxiety score was associated with being older. Relationship to patient was also a significant factor within the model; being a parent to the patient significantly predicted anxiety compared with being the adult child of the patient. The final model demonstrated good fit to the data (*F* = 3.507, *p* < .001) and explained 33% of the variation in anxiety symptoms (*R*^2^ = .327). The significant variables accounted for approximately 23% of the variation in anxiety symptoms (adjusted *R*^2^ = .233).

**Table 3.** Linear regression results

	Bivariate model	Model with covariates	Partnered or married subsample
Variables	b (SE)	b (SE)	b (SE)
Intercept	73.307***	77.457***	91.274***
	(2.869)	(4.926)	(7.412)
**Family interactions**	−0.288***	−0.212***	−0.175*
	(0.049)	(0.059)	(0.068)
**Age**		−0.157*	−0.126
		(0.061)	(0.066)
**Race (Reference = White)**			
Black/African American		−2.325	−2.192
		(2.257)	(2.871)
Other race		1.953	2.820
		(2.380)	(2.727)
**Ethnicity (Reference = Not Hispanic or Latina/o)**			
Hispanic or Latina/o		−0.229	0.583
		(3.305)	(3.466)
**Sex (Reference = Male)**			
Female		5.613***	6.944***
		(1.341)	(1.445)
**Marital status (Reference = Single, never married)**			
Married		6.406*	
		(2.819)	
In a committed partnership		7.792* (3.088)	
Divorced or separated		6.037	
		(3.465)	
Widowed		7.151	
		(4.956)	
Other		4.096	
		(6.743)	
**Caregiver relationship to patient (Reference = Adult child)**			
Spouse		−0.162	0.990
		(1.976)	(2.078)
Ex-spouse		−4.358	0.678
		(4.819)	(9.677)
Unmarried partner		0.897	2.998
		(3.193)	(2.809)
Parent		−7.078*	−5.385
		(2.996)	(3.384)
Sibling		−1.750	−0.724
		(2.849)	(3.687)
In-law		−3.481	−3.389
		(4.498)	(4.953)
Other family member		−1.610	5.878
		(3.767)	(5.200)
**Employment status (Reference = Unemployed)**			
Employed part-time		−0.898	−3.567
		(2.795)	(3.153)
Employed full-time		−1.046	−3.529
		(2.469)	(2.756)
Retired		0.430	−3.231
		(2.693)	(3.022)
Other		−2.247	−4.244
		(2.944)	(3.673)
**Household income (Reference = Less than $20,000/year)**			
$20,000–$40,000/year		−4.331	−8.820*
		(2.686)	(3.384)
$40,001–$70,000/year		−5.134	−11.563***
		(2.703)	(3.230)
$70,000+/year		−3.669	−8.746**
		(2.466)	(2.945)
**Social support**		−0.095	−0.184*
		(0.068)	(0.086)
**Relationship satisfaction (Reference = Very dissatisfied)**			
Somewhat dissatisfied			−1.714
			(5.936)
Neither dissatisfied nor satisfied			−1.003
			(5.799)
Somewhat satisfied			−0.331
			(5.116)
Very satisfied			−1.835
			(4.817)
No. obs.	244	215	173
*R* squared	0.125	0.327	0.370
Adjusted *R* squared	0.122	0.233	0.263
*F* statistic	34.662	3.507	3.457
*P* value	<0.001	<0.001	<0.001
AIC	1765.276	1543.492	1233.364

**Note**: SE = standard error. obs = observations. AIC = Akaike information criterion.

*** *p* < .001; ** *p* < .01; * *p* < .05.

Model results for our married and partnered subsample indicated that perceptions of the quality of family interactions were significantly associated with symptoms of anxiety (*b* = −.175, SE = .068). Sex and income levels were also significantly associated with anxiety. Being female was in the same direction as in the full model with covariates. Household income greater than $20,000 per year was associated with lower anxiety scores on average compared to household income of less than $20,000 per year. Model results did not show a significant association between relationship satisfaction and anxiety symptoms among married or partnered caregivers. However, unlike in the full analytic sample, among married and partnered caregivers, social support was found to be significantly associated with anxiety (*b* = −.184, SE = .086). The estimate indicates that for every 10-point increase in social support from friends and significant others, anxiety symptoms decreased by 1.8 points. The relationship satisfaction model demonstrated a good fit to the data (*F* = 3.457, *p* < .001) and explained about 37% of the variation in anxiety (*R*^2^ = .370). The statistically significant variables accounted for about 26% of the variation in anxiety (adjusted *R*^2^ = .263).

## Discussion

This study aimed to determine the relationship between perceptions of the quality of family interactions and symptoms of anxiety among caregivers of people living with cancer. Our hypothesis was supported by the finding of a negative relationship between these variables. This relationship has been previously described in qualitative literature (Taleghani et al. 2021), which provides rich, in-depth description of tensions within the cancer patient’s family and their effects on cancer caregivers. Our study strengthens the literature via its quantitative approach utilizing validated instrumentation to examine this association. It also highlights the significant potential of family-oriented interventions – of which there are very few (Oliveira et al. 2022) – to reduce caregiver anxiety.

Our study provides additional perspective on the experiences of married and partnered caregivers in juxtaposition to caregivers who are not in a committed relationship. While much of the literature describes marriage as a protective factor for wellbeing (Jace and Makridis 2021; Kravdal et al. 2023) and quality of life (Huntington et al. 2022; Tatangelo et al. 2017), married or partnered caregivers in our study, on average, reported higher anxiety scores than single participants. This may be attributable, in part, to the fact that many cancer aregivers were married or partnered to the person with cancer for whom they were caring, which has been shown in some studies to correlate to worse caregiver mental health (Shaffer et al. 2017). Although the literature describes a well-documented link between marital quality and mental health (Kiecolt-Glaser and Wilson 2017; Wilson and Marini 2023), we did not identify an association between relationship satisfaction and caregiver anxiety in our sub-analysis of married or partnered caregivers. This is possibly due to limited variability in responses to our relationship satisfaction question. In addition, we note that we used a single, researcher-generated item to assess relationship satisfaction due to constraints of questionnaire length; future studies should evaluate this further with more comprehensive, validated instrumentation, given the discrepancy between our findings and prior research.

Interestingly, among our married or partnered subsample (but not amongst the full sample), caregiver’s household income was significantly associated with anxiety symptoms. Caregivers in households with lower incomes had worse anxiety scores. It is unclear from this quantitative study why this is the case; however, it may be that married or partnered caregivers face higher financial stress related to caregiving for a spouse or partner. Other scholars have described that caregivers of cancer patients face reduction in working hours, decreases in income, worsened debt, and loss of employment following their assumption of the caregiver role (Bradley et al. 2023; Natvig et al. 2021); even more concerning is that these financial impacts are inequitably borne out by lower income caregivers and female caregivers (Bradley et al. 2023). Given that lower income caregivers have less access to both paid sick leave and paid family and medical leave due to variance in laws between states (Saad-Lessler and Bahn 2024), it is likely that lower income caregivers have less buffer to weather these impacts which may place more strain on mental health. Caregivers who reduce their hours also report worse anxiety compared to those who do not reduce their hours (Natvig et al. 2021). Future studies should assess the nuances of the roles of income and socioeconomic status on caregiver anxiety symptoms and consider targeted interventions for high-risk groups to move toward equitable mental health outcomes for caregivers of cancer patients.

Among married or partnered caregivers in our study sample, social support was significantly associated with less anxiety, which is consistent with findings reported from several trials of social support interventions (Badger et al. 2020; Carr et al. 2023; Trevino et al. 2021). Spouses and partners provide significant support to their partners ((Bierman et al. 2023). Our finding may be due to a large proportion of the married/partnered provided care for their spouse/partner. Having a spouse with cancer likely changes the nature of the support available to the caregiver, thus making support from outside social support paramount to maintaining mental wellness. Taken together, these results point to the need for additional research focused on understanding the likely complex relationship between caregivers’ marital/relationship status and their emotional wellbeing.

The relationship between patient and caregiver likely plays a significant role in one’s caregiving experience (Herbst et al. 2020) and mental health, yet the literature thus far has predominantly focused on spousal caregivers and/or adult–child caregivers to parents with cancer leaving little to be known about parents caring for ill adult children. Our study starts to fill this gap; we found that there was a significant difference in anxiety symptoms for adult children caring for their ill parent compared to a parent caring for their ill adult child. Being a parent caregiver to an adult child with cancer was associated with less anxiety. To the authors’ knowledge, this is the first quantitative study demonstrating a difference in anxiety symptoms between parent and adult–child caregivers to cancer patients. This finding is consistent with the life course literature which has demonstrated that older adults have generally improved emotional wellbeing as they shift their goals toward prioritization of emotional regulation and positive life experiences (Lutz and Van Orden 2020). It is also possible that this finding may be due to underreporting of anxiety symptoms by older adults; there is a cultural misconception that some of the symptoms of strained mental health are a normal and expected part of the aging (Lutz and Van Orden 2020) which may lead respondents to under-report symptoms. Additionally, it is likely that parental caregivers to ill adult children have different experiences than adult child caregivers to ill parents. A recent qualitative study by Breuning et al. (2024) examined differences in psychosocial burdens based on relationship to cancer patient; they found that parents of ill adult children reported different types of difficulties including dealing with their own emotions without burdening the sick adult child and stress due to time burden; unlike adult children caring for a sick parent, these parental caregivers did not report the same degree of changes to daily life routine due to the adult child’s cancer diagnosis. While this qualitative work is somewhat limited in ability to add to our understanding of why this current work suggests parental caregivers of an ill adult child may have less anxiety, when combined with our findings, it is clear that nuances based on caregiver–patient relationship do exist. These findings amplify the need for further study.

Finally, it is also possible that adult children caring for an ill parent have additional strain placed on their families in the wake of the parent’s illness, contributing to worsened anxiety symptoms. With shifts in workforce demands on families, many younger adults rely heavily on their parents to provide childcare to their children (Geurts et al. 2015); the loss of the grandparent role, families may face additional challenges in finding childcare. It is possible that many of the participants in our study are part of this “sandwich generation,” marked by the additive stress, emotional, and financial challenges of caring for an ill parent while simultaneously caring for their children (Lei et al. 2023). Our study adds new information on differences based on relationship to the cancer patient and first steps in identification of differential risks for certain groups. Specific caregivers may benefit from targeted interventions; additional quantitative and qualitative studies are needed to elucidate the nuances of the parent–child relationship for caregivers of cancer patients.

### Study limitations

Numerous study limitations should be noted. We collected cross-sectional, self-reported data, limiting our ability to comment on causality or directionality. This is particularly important, given that the relationship between caregivers’ perceptions of the quality of their family interactions and their symptoms of anxiety are likely bidirectional. That is, dissatisfying or conflictual family interactions could lead to feelings of anxiety and, conversely, feelings of anxiety could result in family interactions of poorer quality. Additionally, the sample included mostly caregivers from socially advantaged groups, including higher numbers of married or partnered participants, employed participants, and those with higher incomes. Given our finding that, among married or partnered caregivers, income is associated with symptoms of anxiety, it is important to continue to study individuals from socially disadvantaged groups, including caregivers with lower incomes. Furthermore, given the substantial literature describing health disparities and inequity in cancer care (Institute of Medicine 2003; Meints et al. 2019; Sorice et al. 2022), it is possible that the experiences of socially disadvantaged family caregivers may be different than those reported here as our sample was predominately White and non-Hispanic. Additionally, our study included mostly cisgender, heterosexual couples. Given increased awareness of the disparate experiences of people with serious illness with marginalized sexual orientation and gender identities (Candrian et al. 2021; Kortes-Miller et al. 2018; Maingi et al. 2018), it is possible that the experiences of these groups may be different. Future studies with rigorous longitudinal assessments of diverse groups are needed.

## Conclusion

This study provides new insight into the relationship between caregiver’s family interactions and their anxiety symptoms. We found that caregivers with negative family interactions have higher anxiety symptoms. Additionally, we identified unique subpopulations including those who are married or in a committed partnership and cancer caregivers to their adult children that may benefit from targeted interventions. Future studies are needed to better understand the nuanced experiences of these caregivers. Clinicians, support network members, and policy makers will benefit from these findings by enabling them to ask more informed questions of caregivers of cancer patients to provide tailored resources and positive coping skills development.

## References

[ref1] Ahmad Zubaidi ZS, Ariffin F, Oun CTC, et al. (2020) Caregiver burden among informal caregivers in the largest specialized palliative care unit in Malaysia: A cross sectional study. *BMC Palliative Care* 19(1), 186. doi:10.1186/s12904-020-00691-1 PMC7722979 33292214

[ref2] Alnahdi GH, Alwadei A, Woltran F, et al. (2022) Measuring family quality of life: Scoping review of the available scales and future directions. *International Journal of Environmental Research & Public Health* 19(23), 15473. doi:10.3390/ijerph192315473 PMC9738839 36497550

[ref3] American Psychiatric Association (2013) *Diagnostic and Statistical Manual of Mental Disorders*, 5th. Arlington, VA: American Psychiatric Association.

[ref4] American Psychological Association (2023) Anxiety. *APA Dictionary of Psychology*. American Psychological Association. https://dictionary.apa.org/.

[ref5] Badger TA, Segrin C, Sikorskii A, et al. (2020) Randomized controlled trial of supportive care interventions to manage psychological distress and symptoms in Latinas with breast cancer and their informal caregivers. *Psychology and Health* 35(1), 87–106. doi:10.1080/08870446.2019.1626395 31189338

[ref6] Benson JJ, Washington KT, Landon OJ, et al. (2023) When family life contributes to cancer caregiver burden in palliative care. *Journal of Family Nursing* 29(3), 275–287. doi:10.1177/10748407231167545 37190779 PMC10330805

[ref7] Bierman A, Lee Y and Penning MJ (2023) Mental health benefits and detriments of caregiving demands: A nonlinear association in the Canadian longitudinal study on aging. *Journal of Aging and Health* 35(5–6), 392–404. doi:10.1177/08982643221125258 36112750 PMC10150259

[ref8] Bradley CJ, Kitchen S and Owsley KM (2023) Working, low income, and cancer caregiving: Financial and mental health impacts. *Journal of Clinical Oncology Official Journal of the American Society of Clinical Oncology* 41(16), 2939–2948. doi:10.1200/JCO.22.02537 37043714 PMC10414725

[ref9] Breuning M, Mählmann S, Kerek-Bodden H, et al. (2024) Family caregivers of cancer patients: Burdens and support preferences of partner, parent, and adult-child caregivers. *Psycho-Oncology* 33(9), e9310–e9318. doi:10.1002/pon.9310 39261295

[ref10] Cai T, Huang Q, Wu F, et al. (2021) Psychometric evaluation of the PROMIS social function short forms in Chinese patients with breast cancer. *Health and Quality of Life Outcomes* 19(1), 149. doi:10.1186/s12955-021-01788-8 PMC8130437 34006304

[ref11] Candrian C, Cloyes KG and Meeks S (2021) “She’s dying and I can’t say we’re married?”: End-of-life care for LGBT older adults. *The Gerontologist* 61(8), 1197–1201. doi:10.1093/geront/gnaa186 33305806

[ref12] Carr AL, Bilenduke E, Adolf E, et al. (2023) A pilot randomized study of a telephone-based cognitive-behavioral stress-management intervention to reduce distress in phase 1 oncology trial caregivers. *Palliative and Supportive Care* 21(5), 820–828. doi:10.1017/S1478951523000196 36994841 PMC10544682

[ref13] Cheng AL, Downs DL, Brady BK, et al. (2023) Interpretation of PROMIS depression and anxiety measures compared with DSM-5 diagnostic criteria in musculoskeletal patients. *JBJS Open Access* 8(1), e22.00110. doi:10.2106/JBJS.OA.22.00110 PMC9872970 36698984

[ref14] Clover K, Lambert SD, Oldmeadow C, et al. (2022) Convergent and criterion validity of PROMIS anxiety measures relative to six legacy measures and a structured diagnostic interview for anxiety in cancer patients. *Journal of Patient-Reported Outcomes* 6(1), 80. doi:10.1186/s41687-022-00477-4 PMC9300804 35857151

[ref15] Cohen S and Wills TA (1985) Stress, social support, and the buffering hypothesis. *Psychological Bulletin* 98(2), 310–357. doi:10.1037/0033-2909.98.2.310 3901065

[ref16] Cothran FA, Paun O, Strayhorn S, et al. (2022) “Walk a mile in my shoes:” African American caregiver perceptions of caregiving and self-care. *Ethnicity & Health* 27(2), 435–452. doi:10.1080/13557858.2020.1734777 32116006 PMC9137429

[ref17] Dambi JM, Corten L, Chiwaridzo M, et al. (2018) A systematic review of the psychometric properties of the cross-cultural translations and adaptations of the Multidimensional Perceived Social Support Scale (MSPSS). *Health and Quality of Life Outcomes* 16(1), 80. doi:10.1186/s12955-018-0912-0 PMC5930820 29716589

[ref18] de Castro NFC, de Melo Costa Pinto R, da Silva Mendonça TM, et al. (2020) Psychometric validation of PROMIS® anxiety and depression item banks for the Brazilian population. *Quality of Life Research* 29(1), 201–211. doi:10.1007/s11136-019-02319-1 31598816

[ref19] De Laurentis M, Rossana B, Andrea B, et al. (2019) The impact of social-emotional context in chronic cancer pain: Patient-caregiver reverberations: Social-emotional context in chronic cancer pain. *Supportive Care in Cancer* 27(2), 705–713. doi:10.1007/s00520-018-4530-5 30382393

[ref20] Dsf Y, Cheng S-T and Wang J (2018) Unravelling positive aspects of caregiving in dementia: An integrative review of research literature. *International Journal of Nursing Studies* 79, 1–26. doi:10.1016/j.ijnurstu.2017.10.008 29128685

[ref21] Family Caregiver Alliance (2016) Caregiver statistics: Demographics. https://www.caregiver.org/resource/caregiver-statistics-demographics/ (accessed 13 April 2023).

[ref22] Galbraith M (2005) Prostate cancer survivors’ and partners’ self-reports of health-related quality of life, treatment symptoms, and marital satisfaction 2.5–5.5 years after treatment. *Oncology Nursing Forum* 32(2), E30–E41. doi:10.1188/05.ONF.E30-E41 15759059

[ref60] Geurts T, Van Tilburg T, Poortman A-R , et al. (2015) Child care by grandparents: Changes between 1992 and 2006. *Ageing and Society* 35(6), 1318–1334. doi:10.1017/s0144686x14000270

[ref23] Hastert TA, Ruterbusch JJ, Nair M, et al. (2020) Employment outcomes, financial burden, anxiety, and depression among caregivers of African American cancer survivors. *JCO Oncology Practice* 16(3), e221–e233. doi:10.1200/JOP.19.00410 31496392 PMC7069702

[ref24] Herbst FA, Gawinski L, Schneider N, et al. (2020) Adult child-parent dyadic interactions at the end of life: A scoping review. *BMJ Supportive & Palliative Care* 10(2), 175–185. doi:10.1136/bmjspcare-2019-001894 31395611

[ref25] Huntington C, Stanley SM, Doss BD, et al. (2022) Happy, healthy, and wedded? How the transition to marriage affects mental and physical health. *Journal of Family Psychology* 36(4), 608–617. doi:10.1037/fam0000913 34472934 PMC8888778

[ref26] Institute of Medicine (2003) *Unequal Treatment: confronting Racial and Ethnic Disparities in Health Care*. Washington, DC: The National Academies Press. 25032386

[ref27] Jace CE and Makridis CA (2021) Does marriage protect mental health? Evidence from the COVID‐19 pandemic. *Social Science Quarterly* 102, 2499–2515. doi:10.1111/ssqu.13063 PMC8662208 34908604

[ref28] Janson P, Willeke K, Zaibert L, et al. (2022) Mortality, morbidity and health-related outcomes in informal caregivers compared to non-caregivers: A systematic review. *International Journal of Environmental Research & Public Health* 19(10), 5864. doi:10.3390/ijerph19105864 PMC9141545 35627399

[ref29] Kiecolt-Glaser JK and Wilson SJ (2017) Lovesick: How couples’ relationships influence health. *Annual Review of Clinical Psychology* 13(1), 421–443. doi:10.1146/annurev-clinpsy-032816-045111 PMC5549103 28301763

[ref30] Kortes-Miller K, Boulé J, Wilson K, et al. (2018) Dying in long-term care: Perspectives from sexual and gender minority older adults about their fears and hopes for end of life. *Journal of Social Work in End-of-Life & Palliative Care* 14(2-3), 209–224. doi:10.1080/15524256.2018.1487364 30457453

[ref31] Kravdal Ø, Wörn J and Reme B-A (2023) Mental health benefits of cohabitation and marriage: A longitudinal analysis of Norwegian register data. *Population Studies* 77(1), 91–110. doi:10.1080/00324728.2022.2063933 35502948

[ref32] Lei L, Leggett AN and Maust DT (2023) A national profile of sandwich generation caregivers providing care to both older adults and children. *Journal of the American Geriatrics Society* 71(3), 799–809. doi:10.1111/jgs.18138. 36427297 PMC10023280

[ref33] Litzelman K and Al Nassar N (2022) Partner effects on caregiver and care recipient depressed mood: Heterogeneity across health condition and relationship type. *Journal of Behavioral Medicine* 45(5), 750–759. doi:10.1007/s10865-022-00343-0 35907099 PMC10202032

[ref34] Lutz J and Van Orden KA (2020) Sadness and worry in older adults: Differentiating psychiatric illness from normative distress. *Med Clin North Am* 104(5), 843–854. doi:10.1016/j.mcna.2020.05.001. 32773049 PMC7417641

[ref35] Maingi S, Bagabag AE and O’Mahony S (2018) Current best practices for sexual and gender minorities in hospice and palliative care settings. *Journal of Pain and Symptom Management* 55(5), 1420–1427. doi:10.1016/j.jpainsymman.2017.12.479 29288882

[ref36] Meints SM, Cortes A, Morais CA, et al. (2019) Racial and ethnic differences in the experience and treatment of noncancer pain. *Pain Management* 9(3), 317–334. doi:10.2217/pmt-2018-0030 31140916 PMC6587104

[ref37] National Alliance for Caregiving and AARP (2020) *Caregiving in the U.S. 2020 Age 50+*. Washington, DC: AARP, https://www.aarp.org/content/dam/aarp/ppi/2021/05/caregiving-in-the-united-states-50-plus.doi.10.26419-2Fppi.00103.022.pdf (accessed 11 June 2024).

[ref38] National Cancer Institute, NIH, DHHS (2023) Cancer trends progress report, Bethesda, MD: National Cancer Institute. https://progressreport.cancer.gov.

[ref39] Natvig C, Mikulich-Gilbertson SK, Laudenslager ML, et al. (2021) Association between employment status change and depression and anxiety in allogeneic stem cell transplant caregivers. *Journal of Cancer Survivorship: Research and Practice* 16(5), 1090–1095. doi:10.1007/s11764-021-01099-3 34417708 PMC9192097

[ref40] Nissen KG, Trevino K, Lange T, et al. (2016) Family relationships and psychosocial dysfunction among family caregivers of patients with advanced cancer. *Journal of Pain and Symptom Management* 52(6), 841–849.e1. doi:10.1016/j.jpainsymman.2016.07.006 PMC5497710 27521285

[ref41] Ochoa C, Buchanan Lunsford N and Lee Smith J (2020) Impact of informal cancer caregiving across the cancer experience: A systematic literature review of quality of life. *Palliative and Supportive Care* 18(2), 220–240. doi:10.1017/S1478951519000622 31588882 PMC8678891

[ref42] Oliveira C, Fonseca G, Areia NP, et al. (2022) Caring for people who take care: What is already done? *Palliative and Supportive Care* 20(5), 720–730. doi:10.1017/S147895152100119X 36111734

[ref43] Oliver DP, Demiris G, Washington KT, et al. (2017) Challenges and strategies for hospice caregivers: A qualitative analysis. *The Gerontologist* 57(4), 648–656. doi:10.1093/geront/gnw054 27048707 PMC5881722

[ref44] Pilkonis PA, Choi SW, Reise SP, et al. (2011) Item banks for measuring emotional distress from the Patient-Reported Outcomes Measurement Information System (PROMIS®): Depression, anxiety, and anger. *Assessment* 18(3), 263–283. doi:10.1177/1073191111411667 21697139 PMC3153635

[ref45] Purvis TE, Neuman BJ, Riley LH, et al. (2019) Comparison of PROMIS anxiety and depression, PHQ-8, and GAD-7 to screen for anxiety and depression among patients presenting for spine surgery. *Journal of Neurosurgery: Spine*, 30(4), 1–8. doi:10.3171/2018.9.SPINE18521 30660113

[ref46] R Core Team (2021) R: A language and environment for statistical computing. (Version 4.3.0), Vienna, Austria: R Foundation for Statistical Computing. https://www.R-project.org.

[ref47] Rettig KD and Leichtentritt RD (1999) A general theory for perceptual indicators of family life quality. *Social Indicators Research* 47(3), 307–342. doi:10.1023/A:1006837329353

[ref48] Rose KM, Williams IC, Anderson JG, et al. (2021) Development and validation of the Family Quality of Life in Dementia scale. *The Gerontologist* 61(6), e260–e268. doi:10.1093/geront/gnaa022 32329513

[ref49] Rurka M, Jill Suitor J and Gilligan M (2021) The caregiver identity in context: Consequences of identity threat from siblings. *The Journals of Gerontology: Series B* 76(8), 1593–1604. doi:10.1093/geronb/gbaa099 PMC8436691 32674158

[ref50] Saad-Lessler J and Bahn K (2024) *The Importance of Paid Leave for Caregivers: Labor Force Participation Effects on California’s Comprehensive Paid Family and Medical Leave*. https://www.americanprogress.org/article/importance-paid-leave-caregivers/ (accessed 11 June 2024).

[ref51] Shaffer KM, Jacobs JM, Nipp RD, et al. (2017) Mental and physical health correlates among family caregivers of patients with newly-diagnosed incurable cancer: A hierarchical linear regression analysis. *Supportive Care in Cancer* 25(3), 965–971. doi:10.1007/s00520-016-3488-4 27866337 PMC5269509

[ref52] Sorice KA, Fang CY, Wiese D, et al. (2022) Systematic review of neighborhood socioeconomic indices studied across the cancer control continuum. *Cancer Medicine* 11(10), 2125–2144. doi:10.1002/cam4.4601 35166051 PMC9119356

[ref53] Starr LT, Bullock K, Washington K, et al. (2022) Anxiety, depression, quality of life, caregiver burden, and perceptions of caregiver-centered communication among black and white hospice family caregivers. *Journal of Palliative Medicine* 25(4), 596–605. doi:10.1089/jpm.2021.0302 34793244 PMC8982115

[ref54] Taleghani F, Ehsani M, Farzi S, et al. (2021) Challenges to family caregivers in caring for gastric cancer patients from perspectives of family caregivers, patients, and healthcare providers: A qualitative study. *Indian Journal of Palliative Care* 27, 521–529. doi:10.25259/IJPC_98_21 34898947 PMC8655636

[ref55] Tatangelo G, McCabe M, Campbell S, et al. (2017) Gender, marital status and longevity. *Maturitas* 100, 64–69. doi:10.1016/j.maturitas.2017.03.002 28539178

[ref56] Trevino KM, Stern A, Hershkowitz R, et al. (2021) Managing anxiety from cancer (MAC): A pilot randomized controlled trial of an anxiety intervention for older adults with cancer and their caregivers. *Palliative and Supportive Care* 19(2), 135–145. doi:10.1017/S1478951521000286 33818370 PMC8085071

[ref57] Victorson D, Schalet BD, Kundu S, et al. (2019) Establishing a common metric for self-reported anxiety in patients with prostate cancer: Linking the memorial anxiety scale for prostate cancer with PROMIS anxiety. *Cancer* 125(18), 3249–3258. doi:10.1002/cncr.32189 31090933

[ref58] Wilson SJ and Marini CM (2023) The days add up: Daily marital discord and depressive reactivity linked to past-month depressed mood and marital risk across 10 years. *Journal Of Social & Personal Relationships* 40(4), 1172–1193. doi:10.1177/02654075221116277 37457374 PMC10348706

[ref59] Zimet GD, Powell SS, Farley GK, et al. (1990) Psychometric characteristics of the Multidimensional Scale of Perceived Social Support. *Journal of Personality Assessment* 55(3–4), 610–617. doi:10.1080/00223891.1990.9674095 2280326

